# Current Insights Regarding Intestinal Failure-Associated Liver Disease (IFALD): A Narrative Review

**DOI:** 10.3390/nu15143169

**Published:** 2023-07-17

**Authors:** Marija Zafirovska, Aleksandar Zafirovski, Nada Rotovnik Kozjek

**Affiliations:** 1 Faculty of Medicine, University of Ljubljana, Vrazov trg 2, 1000 Ljubljana, Slovenia; aleksandar.zafirovski@sb-je.si (A.Z.); nada.rotovnik-kozjek@mf.uni-lj.si (N.R.K.); 2 Association of General Practice/Family Medicine of South-East Europe (AGP/FM SEE), St. Vladimir Komarov No. 40/6, 1000 Skopje, North Macedonia; 3 General Hospital Jesenice, Cesta Maršala Tita 112, 4270 Jesenice, Slovenia; 4 Clinical Institute of Radiology, University Medical Centre Ljubljana, Zaloška Cesta 7, 1000 Ljubljana, Slovenia; 5 Department for Clinical Nutrition, Institute of Oncology Ljubljana, Zaloška Cesta 2, 1000 Ljubljana, Slovenia

**Keywords:** intestinal failure, intestinal failure associated liver disease, parenteral nutrition

## Abstract

Intestinal failure-associated liver disease (IFALD) is a spectrum of liver disease including cholestasis, biliary cirrhosis, steatohepatitis, and gallbladder disease in patients with intestinal failure (IF). The prevalence of IFALD varies considerably, with ranges of 40–60% in the pediatric population, up to 85% in neonates, and between 15–40% in the adult population. IFALD has a complex and multifactorial etiology; the risk factors can be parenteral nutrition-related or patient-related. Because of this, the approach to managing IFALD is multidisciplinary and tailored to each patient based on the etiology. This review summarizes the current knowledge on the etiology and pathophysiology of IFALD and examines the latest evidence regarding preventative measures, diagnostic approaches, and treatment strategies for IFALD and its associated complications.

## 1. Introduction

Intestinal failure (IF) is the state where the functionality of the gut falls below the minimum level necessary for the absorption of nutrients and/or water and electrolytes. In such cases, intravenous supplementation becomes necessary to sustain health and/or growth [1]. According to disease duration and stability, IF has been classified into three categories:

Type I: acute condition, usually after abdominal surgery or during critical illness; patients require short-term parenteral nutrition and/or fluid support lasting for days.
Type II: prolonged acute condition, in which patients are metabolically unstable and require multidisciplinary care lasting for week to months.
Type III: chronic condition, in which can be reversible or irreversible; patients are metabolically stable and require long-term parenteral nutrition over months or years [2,3].

Parenteral nutrition (PN) is a life-saving therapy for individuals with IF, as it is delivers vital nutrients directly into the bloodstream, thereby circumventing the digestive system when oral or enteral nutrition (EN) options are inadequate or restricted [4]. However, the inappropriate administration of PN has been linked to numerous complications including mechanical (thromboembolism), infectious (central line-associated blood stream infections), and metabolic (hyperglycemia, refeeding syndrome, metabolic bone disease, abnormal liver function tests, cholelithiasis) [5,6]. Intestinal failure-associated liver disease (IFALD) is one of the most serious metabolic complications of the inadequate use of PN because of its complex etiopathogenesis, including different pathophysiological mechanisms of intestinal failure. Therefore, the term IFALD has superseded the previous designations of parenteral nutrition-associated liver disease (PNALD) and parenteral nutrition-associated cholestasis (PNAC) due to advances in understanding that properly administered and balanced parenteral nutrition does not cause liver damage and further research into other causes of the condition [7,8].

IFALD develops in patients with IF due to a complex and multifactorial etiology; it consists of a range of liver diseases that include cholestasis, biliary cirrhosis, steatohepatitis, and gallbladder disease [9,10]. Gallbladder disease related to IFALD is not specifically covered in this review.

This review aims to provide a comprehensive overview of the present understanding regarding the etiopathogenesis of IFALD and to examine the most relevant evidence regarding preventive, diagnostic, and treatment strategies for IFALD and its associated complications.

## 2. Methods

### 2.1. Selection Criteria

The eligibility criteria for performing the search and selection of studies are shown in the table below (See Table 1).

### 2.2. Search Strategy

An extensive literature search was performed in PubMed, Embase, Medline, and the Cochrane library to identify eligible studies. The search was repeated twice, with the last search being performed in July 2022. Additional searching of the grey literature or hand searching were not performed.

The search terms used in PubMed were as follows: (liver disease OR liver dysfunction OR liver dysfunctions) AND ((parenteral nutrition) OR (intestinal failure) AND (humans [Filter])). For Embase and Medline, we used the following: {[(intestinal failure.mp. or exp intestinal failure) OR (exp home parenteral nutrition/or exp parenteral nutrition)] AND (liver disease.mp. or exp liver disease)}.

All the search results were summed up and duplicates were removed. For the first selection, two reviewers screened potentially relevant articles based on the title and abstract according to predefined eligibility criteria. For the second selection, full articles were read to determine further inclusion. Cross-references of relevant studies and reviews were screened.

## 3. Epidemiology

The prevalence of IFALD demonstrates notable variation across studies, with reported ranges of 40–60% in the pediatric population, up to 85% in neonates, and between 15–40% in the adult population [8,11]. Among a cohort of 302 children receiving home PN between 1980 and 1999, IFALD was observed in 23% of cases, while only seven patients (2%) experienced fatal liver failure and seven patients (2%) underwent liver–small bowel transplantation [12]. During a 6-year follow-up of 90 adults receiving home PN between 1985 and 1996, a separate study revealed that 50% of the participants developed persistent IFALD [13]. A one-year prospective study on adults indicated that IFALD can occur at the beginning of PN; however, IFALD-cholestasis and IFALD-fibrosis may disappear during the initial stage of PN, while IFALD-steatosis persists [14]. In a recent retrospective study on 279 hospitalized children receiving long-term PN, 22% developed IFALD and 4% progressed to end-stage liver disease. IFALD was associated with younger age, longer treatment, longer hospitalization, surgical diagnosis, and prematurity [15]. The length of PN has been found to have a direct correlation with the incidence of PNAC, as demonstrated by a systematic review. The overall incidence of PNAC was 28.2% and that of IFALD 49.8% in pediatric patients [16]. Mortality as high as 40% has been reported in patients with established IFALD, representing the main indication for intestinal transplantation in children [17,18].

IFALD can proceed to fibrosis or possibly cirrhosis, and can cause severe morbidity and mortality; therefore, it poses a risk to patients with chronic IF. Across various studies, 3.6–8.8% of deaths among patients with PN-dependency and benign underlying disease were because of IFALD [19,20,21].

## 4. Etiology and Pathophysiology

### 4.1. Risk Factors

The table presented below (See Table 2) provides a summary of the diverse risk factors associated with IFALD within both the adult and pediatric populations.

### 4.2. Intravenous Lipid Emulsions

In order to provide a concentrated source of non-protein energy, PN is administered alongside an intravenous lipid emulsion (ILE). The first-generation ILE, which was based on soybean oil, was introduced in 1961 by Arvid Wretlind. Subsequently, four generations of oil-based ILEs have been developed from different combinations of various oils, such as soybean, coconut, safflower, olive, or fish oil. The fourth generation includes Omegaven^®^ 10%, Lipiderm LipoPlus^®^ 20%, and SMOFlipid^®^ 20% [35].

Soybean oil-based ILE (SOLE) and safflower oil-based ILE both have a high concentration of ω-6 polyunsaturated fatty acids (PUFAs) and low concentration of ω-3 PUFAs. The ratio of linoleic acid (LA) to α-linoleic acid (ALA) in SOLE is 7:1. Safflower oil-based ILE has higher concentration of LA and lower concentration of ALA compared to SOLE [4,22]. Because both are plant-derived, they contain a high concentration of phytosterols, which are not absorbed in the gastrointestinal tract and tend to accumulate in the hepatocytes when given intravenously [37,38]. Soybean oil contains γ-tocopherol, a less bioavailable form of vitamin E [39].

The ILE used in PN often contains medium-chain triglycerides (MCTs) derived from coconut oil, specifically capric and caprylic acids, which typically have 6–12 carbon atoms. MCTs are protein-sparing, easily metabolized, do not accumulate in the liver, and are peroxidation-resistant. However, the sole use of coconut oil is not favourable, as it lacks essential fatty acids, which can potentially lead to the development of Essential Fatty Acid Deficiency (EFAD) in patients. Sole use of olive oil can lead to the same condition, as it contains approximately 5% LA and less than 1% ALA. However, due to the presence of monounsaturated fatty acids (MUFA) such as oleic acid, olive oil is less inflammatory compared to SOLE [40].

Fish oil exhibits a high content of α-tocopherol, low levels of phytosterols, and a substantial concentration of the anti-inflammatory ω-3 fatty acids eicosapentaenoic acid (EPA) and docosahexaenoic acid (DHA) [4,40]. Fish oil-based ILE (FOLE) facilitates the resolution of cholestasis and effectively supports growth and development while avoiding an elevated risk of EFAD [41]. FOLE can reverse IFALD, increase survival, and avoid the need for transplantation [42].

Mixed-oil or composite ILE (MOLE) options are superior to ILEs based only on ω-6 derived from soybean. They are formulated with a mixture of soybean oil to provide essential fatty acids, coconut oil rich in medium chain triglycerides, olive oil rich in monounsaturated fatty acids, and fish oil for ω-3 fatty acids [43]. Composite emulsions have been shown to reduce the frequency of inflammation, cholestasis, and hyperbilirubinemia. In fact, composite emulsions appear to be superior to conventional ILEs at regular dosages for neonatal growth in the overall neonatal population [44].

### 4.3. Nutrient Toxicity

#### 4.3.1. Glucose Overload

Glucose infusion rates greater than 5 mg/kg/min cause steatosis by boosting insulin release, which in turn stimulates hepatic lipogenesis and acylglycerol synthesis, and by blocking mitochondrial fatty acid oxidation, which leads to a buildup of TAG in hepatocytes [9]. It has been demonstrated that allowing ≥8 h per day without parenteral glucose infusion lowers insulin levels and improves liver function tests [45]. Based on glucose oxidation rates, the American Society for Parenteral and Enteral Nutrition guidelines suggest limiting glucose infusion rate to 12–14 mg/kg/min in infants and young children, 8–10 mg/kg/min in adolescents, and 4–5 mg/kg/min in adults [46].

#### 4.3.2. ω-6 vs. ω-3 Fatty Acids

The major pro-inflammatory substances in SOLE are ω-6 fatty acids, which act as precursors for arachidonic acid, which, through the cyclooxygenase and lipoxygenase pathways lead to synthesis of prostanoids and leukotrienes [35]. A pro-inflammatory environment causes liver injury, and when SOLE is given at doses greater than 1 g/kg body weight this is even more pronounced [42,47].

ω-3 fatty acids, on the other side, are beneficial due to their anti-inflammatory characteristics. Several G-protein-coupled receptors (GPR), such as GPR 12, are receptors for ω-3 fatty acids found in fish oil. Recent research has shown that several of the therapeutic advantages of ω-3 fatty acids in organs such the liver, brain, and bones are mediated by their interaction with these receptors [48,49]. According to one meta-analysis, the outcomes of patients with sepsis were improved by PN supplemented with ω-3 fatty acids [50].

In infants and children with IFALD, it has been shown that cholestasis developed on SOLE can be reversed by substituting it with a limited duration of FOLE [41,51,52,53]. Another study suggested that the quantity of lipids, rather than the specific lipid type, influence cholestasis, as in infants the direct bilirubin levels were normalized equally well with low doses of FOLE and SOLE (1 g/kg/day) while showing no differences in nutritional status [47]. Several randomized trials have shown that the reduction of SOLE to <1 g/kg/day effectively reverses IFALD without having to change the composition of the ILE [54,55].

#### 4.3.3. Medium Chain Triglycerides (MCTs)

MCTs are triacylglycerols with 6–12 carbon saturated fatty acids; they are mainly derived from coconut and palm kernel oils. They are 100 times more soluble than LCTs, and as such they are quickly hydrolyzed and absorbed from the intestinal lumen. They are metabolized quickly, and do not accumulate in the liver due to their ability to enter mitochondria without the need for carnitine and their reduced susceptibility to the influence of glucose and insulin [56]. No human studies were found on the effects of MCTs on IFALD. Animal models have shown that MCTs have an anti-inflammatory effect [57,58] as well as a protective role in alcohol liver injury [59] and NAFLD [60].

#### 4.3.4. Phytosterols

Phytosterols are plant-derived sterols that are structurally similar to mammalian cholesterol. Sitosterol, campesterol, and stigmasterol are the main phytosterols found in SOLE and MOLE [39]. Because most ILEs are based on vegetable oils, during PN the amount of phytosterols in the blood increases; they accumulate in the liver and inhibit acid bile transport [61,62]. There are two theories about how phytosterols may induce cholestasis:

1. Disruption of the FXR-FGF19 signaling axis. One of the most prevalent phytosterols, stigmasterol is an antagonist to the farnesoid X receptor and reduces the expression of the genes for bile acid transporters. The antagonistic effect of stigmasterol has been found to be dose-dependent and to involve competition at the FXR ligand-binding domain [63].
2. Enhancement of the inflammatory response in macrophages. In animal models, it has been shown that phytosterols maximize the inflammatory response in Kupffer cells synergistically with lipopolysaccharides (LPS) [62,64]. A study on a mouse model supported the hypothesis that IFALD results from proinflammatory activation of hepatic macrophages by intestinal derived LPS and circulating phytosterols. This leads to generation of IL-1β, which binds to the receptors on the hepatocytes and increases NF-κB translocation to the nucleus, in turn leading to suppression of genes encoding FXR and LXR as well as FXR-regulated and LXR-regulated genes [65].

Several studies have shown an association of phytosterol levels with IFALD in patients receiving PN [32,33,34]. Serum phytosterol levels histologically reflect the liver injury in IFALD [66]. In a number of small studies, it has been demonstrated that switching from high-phytosterol ILEs to those with low-phytosterol content can improve the resolution of IFALD. However, prospective randomized blinded clinical trials with long durations that separate the impact of phytosterols from other components of the ILE are necessary in order to ascertain whether phytosterols in ILEs are a contributing factor to IFALD [38].

#### 4.3.5. Trace Elements Overload

Copper is an essential trace element involved in different processes as an enzymatic cofactor, and around 80% is excreted through the bile [67]. In a study that included 28 adult patients IFALD and 10 adult patients with drug-induced cholestatic liver disease, it was shown that PN can lead to copper accumulation in the liver due to disruptions in the bile acid pool. While the levels of copper in the liver were not associated with the duration of PN, patients with chronic cholestasis accumulated more copper, which leads to liver damage [68]. In preterm infants there is an increased copper requirement, low levels of ceruloplasmin, and reduced biliary excretion of copper, which might lead to accumulation in the hepatocytes. While the toxic dose of parenteral copper is undefined, one study found that copper supplementation of 20 μg/kg/day did not lead to worsening of liver disease in cholestatic preterm infants [69]. Copper can additionally be present as a contaminant in certain components of PN, including amino acids and sterile water [70]. Containers frequently include aluminium, which may seep into stored IVLE. A study on a rat model has shown that aluminium exposure contributes to PN-induced hepatobiliary dysfunction and steatosis [31]. Adult patients taking PN may occasionally develop aluminium toxicity if they have risk factors such as kidney disease [30]. Hypermanganesemia has been reported in patients receiving PN [29]. The biliary system is responsible for excreting 90% of manganese, and manganese overload may cause cholestasis [28]. Di (2-Ethylhexyl) Phthalate (DEHP), which is found in PVC infusion systems, may contribute to cholestasis and fibrosis [27]. A study on infants showed that switching from PVC to non-PVC infusion sets resulted in a substantial reduction in cholestasis (from 50% to 13%) in newborns receiving PN [26].

### 4.4. Nutrient Deficiency

#### 4.4.1. Choline, Carnitine, N-Acetylcysteine

It has been shown that lack of choline leads to hepatic morphologic and hepatic aminotransferase abnormalities; however, current PN are not supplemented with it. Choline deficiency has been proven to contribute to IFALD in both adults [71] and infants [72]. Adults on PN with IFALD who received choline supplementation over 24 weeks showed reduction in the hepatic markers ALT, AST, and ALP [71]. Carnitine is not supplemented in current PN either. Carnitine deficiency affects the maturation of the nervous system by causing a reduction in the concentration of ketone byproducts derived from β-oxidation [73], and has been shown to lead to cholestasis [74]. N-acetylcysteine is an essential amino acid that serves as a constituent of the antioxidant glutathione. Low concentrations of N-acetylcysteine are associated with low concentrations of glutathione in preterm infants, which can lead to increased oxidative stress on the hepatocytes [75].

#### 4.4.2. Vitamin E

Vitamin E is an essential nutrient that exists naturally in four isoforms: α, β, γ, and δ-tocopherol, with the most active form being α-tocopherol due to its high affinity for the tocopherol transport protein. Vitamin E has antioxidant effect and is recognized for its ability to regulate the body’s redox balance by protecting cell membranes from oxidative damage [76]. It may be added to ILEs due to its effectiveness in preventing peroxidation in ILEs with high polyunsaturated fatty acids. While SOLE is high in γ-tocopherol and low in α-tocopherol, it is not supplemented with vitamin E because of its 18-carbon fatty acids and the low level of unsaturation. FOLE and MOLE, on the other hand, are supplemented with α-tocopherol because they contain highly unsaturated fatty acids susceptible to oxidation [39]. There were no human studies found that researched the effect on vitamin E in preventing IFALD. Studies on animal models showed inconsistent results, with mouse models showing positive effects by preventing steatosis and reducing cytokine response and indicating that the advantages of vitamin E supplementation may be age-dependent [77,78,79,80].

### 4.5. IF-Related Risk Factors

#### 4.5.1. Gut Microbiome

Patients diagnosed with short bowel syndrome (SBS) who experience small intestine bacterial overgrowth (SIBO) and inflammation often encounter disruptions in gut homeostasis and permeability. These disturbances, coupled with intestinal dysbiosis and bacterial translocation, are linked to the development of IFALD [81]. In animal models there is a significant reduction in the ration of Firmicutes to Bacteroidetes [82]. A study showed overabundance of Lactobacilli, Proteobacteria, and Actinobacteria in patients with IF, along with an association between intestinal microbiota and liver steatosis. A possible mechanism is the proinflammatory effects of these bacteria on the intestinal mucosa, which leads to increased permeability and exposure of the liver to gut-derived LPS [83]. In a mouse model, it was shown that LPS can directly activate TLR-4, initiating a pro-inflammatory cascade that contributes to inflammatory and fibrotic alterations of the liver [84]. Alterations in the gut microbiome can lead to increased vulnerability to bacterial invasion due to compromised mucin production by goblet cells as well as a decrease in the levels of antimicrobial lysozyme and defensins [85,86]. D-lactic acidosis and encephalopathy can develop in people with SBS when lactate-producing bacteria in the colon overpopulate [87]. Another study confirmed overabundance of Proteobacteria and decreased level of acetate in SBS infants with IFALD. A newborn with SBS may be more susceptible to LPS absorption into the portal system and activation of the hepatic immune system due to both the intestinal bacterial load during SBS and the bacterial communities already present [88].

#### 4.5.2. Enteral Feeding

PN and insufficient enteral feeding have been related to decreased bactericidal response of Paneth cells, reduced secretion of IgA, heightened gut permeability, and increased translocation of endotoxins and bacteria into the portal circulation leading to hepatocyte inflammation [6]. The absence of nutrients in the gut can lead to intestinal mucosal atrophy and increased permeability, which is related to reduced secretion of intestinal growth factors such as GLP-2. GLP-2 is secreted when bile acids activate the TGR5 receptor in the ileum, and this process is disrupted when the gut function is altered [43,89]. Lack of enteral feeding has been related to bacterial overgrowth due to changes in nutritional availability and decreased intestinal motility [25]. Changes in the gut microbiome during total fasting can result in altered intestinal permeability and the release of pro-inflammatory cytokines by immune cells, leading to the translocation of harmful compounds such as LPS into the liver [90]. Although EN can normalize significant hyperbilirubinemia in PN-dependent neonates, improvements in liver function typically do not start until full EN is tolerated and PN is stopped [91].

#### 4.5.3. Bile Acid Metabolism and Gut–Liver Cross-Talk

The farnesoid X receptor (FXR) is a nuclear receptor that plays a crucial role in regulating bile acid metabolism and homeostasis. It is primarily expressed in the liver and intestine, although it can be found in other tissues as well [92]. The activation of gut nuclear FXR is regulated by bile acids during normal enterohepatic circulation. Ileal enterocytes release the enterokine FGF19 into the portal circulation as a result of the activation of FXR by absorbed bile acids. FGF19 then binds to fibroblast growth factor receptor 4 (FGFR4) on hepatocytes and leads to suppression of CYP7A1, which provides negative feedback on the production of bile acids [93]. In a piglet model, it was shown that decreased circulating FGF19 during total PN may contribute to IFALD [94]. In a prospective study on 52 IF patients on PN, it was observed that the serum levels of FGF19 were nearly three times lower in IF cases compared to the control group. This reduction was particularly evident in patients who had undergone complete resection of the ileum and ileocecal valve, as well as in those with histological evidence of IFALD [24]. The hepatic FXR is more crucial than the intestinal FXR in preventing the synthesis of steroids for bile through the control of CYP8B1. Intestinal and hepatic FXR are both important in FXR-mediated bile acid synthesis, though they differ in the mechanisms by which they repress CYP7A1 and CYP8B1 [95] FXR plays an additional role in glucose homeostasis by suppressing hepatic gluconeogenesis and promoting hepatic glycogen synthesis. FGF19 stimulates protein and glycogen synthesis in the liver, suppresses gluconeogenesis through FoxO1 signaling, and decreases lipogenesis [96,97]. Patients with NAFLD, a condition similar to IFALD, have been found to exhibit increased FoxO1 signaling [98].

#### 4.5.4. Insulin Signaling Pathways

Total PN results in insulin resistance, hyperglycemia, and iatrogenic counter-regulatory hypoglycemia, all of which have a negative impact on the course of treatment. There are three problems that lead to metabolic dysfunction: loss of the physiological incretin effect, loss of the insulinomimetic action of gut-derived peptides, and lipid-induced insulin resistance [99]. When activated by bile acids, the G-coupled protein receptor TGR5 in the gut initiates the release of the metabolic hormones GLP-1 and GLP-2. GLP-1 enhances insulin sensitivity and secretion while regulating hepatic steatosis, whereas GLP-2 regulates gut development and integrity. Serum GLP-2 levels increase proportionally to the length of the resected intestine in pediatric IF patients with small bowel–colic continuity. A decrease in the need for parenteral support has been found to be accompanied by an increase in serum GLP-1 and GLP-2 levels [23].

#### 4.5.5. Metabolomics

Significant alterations in metabolites and pathways, particularly ones related to amino acid metabolism and cell death, were observed in a study involving twenty pediatric patients with SBS on PN support. Patients with SBS and IFALD showed significant reductions in skatole, glabrol, and uridine which were correlated with serum direct bilirubin levels, while SBS patients on long-term PN showed significantly higher glutamine levels. The levels of the tryptophan metabolite skatole had a strong negative correlation with direct bilirubin levels [100]. It has been shown that the dysregulation of the tryptophan metabolism and its different pathways contribute to the progression of liver disease through their effects on the immune response, oxidative stress, and liver inflammation [101].

### 4.6. Systemic-Related Risk Factors

#### 4.6.1. Prematurity

An inverse relationship between birth weight and PNAC was found in a study on 62 premature infants taking PN. Infants with very low birth weight of less than 1 kg had an increased risk of developing cholestasis, with an incidence rate of 50% [102]. Infants receiving PN who were able to initiate enteral feeding within eight days of birth did not have cholestasis. In neonates, the bile acid metabolism is immature and there is a decreased bile acid reservoir, resulting in susceptibility to PNAC [43].

#### 4.6.2. Central Line-Associated Blood Stream Infections (CLABSI) and Sepsis

Sepsis is a notable risk factor for the onset of IFALD. A study on 152 infants receiving PN showed that septic episodes increase the risk of IFALD by 3.2 times [103]. PN requires the insertion of central venous catheters, which can be a major source of bacteriemia and lead to complications such as sepsis. In patients receiving home PN the overall CLABSI rate was 0.87 episodes/1000 PN days. Patients with double-lumen catheters and implanted ports had more CLABSIs; the most common pathogens were Staphylococci [104]. Insufficient enteral feeding additionally contributes to bacterial overgrowth; studies on trauma patients who received enteral feeding showed a lower occurrence of sepsis, intra-abdominal abscesses, pancreatitis, and other infections in comparison to patients on PN [43]. Research has shown that infection and sepsis downregulate FXR receptor activity, which is important for bile acid metabolism [92].

## 5. Diagnosis

### 5.1. Physical Examination

Though findings on physical examination might be normal, they could indicate liver disease with the hallmarks of jaundice, hepatomegaly, and splenomegaly [22,105].

### 5.2. Biomarkers and Imaging

IFALD can be difficult to diagnose, and a perfect non-invasive diagnostic tool has not yet been found. Abnormal liver blood tests are frequently used for patients on PN. Direct bilirubin, ALP, and GGT were shown to be suitable biomarkers in a retrospective study [106]. In another single-center cohort study, GGT, citrulline, and liver stiffness determined by transient elastography (TE) accurately diagnosed active IFALD with diagnostic and follow-up AUROC values >0.90 [107]. However, TE failed to assess the degree of hepatic fibrosis in patients with long-term home PN [108]. On the other hand, acoustic radiation force impulse (ARFI) elastography showed an AUROC of 0.83 and 0.86 in differentiating moderate/severe liver fibrosis from mild disease [109]. Magnetic resonance spectroscopy (MRS) has proven to be feasible for evaluating IFALD. This method can be used to accurately evaluate the degree of steatosis and to monitor the impact of modifications to the PN or ILE composition. ALP levels are often higher in patients with steatosis, while transaminase levels are not increased. MRS could be used prognostically to determine the need for liver biopsy in patients with no clinical signs of advanced IFALD [110]. Proton MRS showed reliable noninvasive assessment of liver fat content in patients receiving long-term PN [111]. Further research is needed to evaluate the effectiveness of possible non-invasive tests compared to liver biopsy for early detection of IFALD.

### 5.3. Liver Biopsy, IFALD Phases, and Liver Histology

Despite its obvious drawbacks, liver biopsy continues to be the most effective method for diagnosing and tracking the development of IFALD.

The hallmark of phase 1 IFALD is cholestasis and inflammation that rapidly advance to fibrosis. The diagnosis is established when infants and children receiving PN for a minimum of 14 days exhibit serum direct/conjugated bilirubin levels >1.0–2.0 mg/dL and >20% of total serum bilirubin and after all other potential causes of cholestasis have been excluded. Liver histology shows cholestasis, bile ductular reaction, portal bile plugs, variable portal inflammation, prominent macrophages, steatosis, and different stages of periportal fibrosis [10].

Phase 2 IFALD occurs following PN termination when steatosis and progressive fibrosis persist.

Currently, there are no widely accepted diagnostic criteria. Liver biopsy may be necessary for diagnosis until less invasive biomarkers are made available [10]. In one study cirrhosis was observed in 18% of pediatric patients with IF [112].

Infants are more likely to have cholestasis than older children or adults, who usually develop steatosis or steatohepatitis. Fibrosis and cirrhosis typically progress more quickly in infants. IFALD may exhibit ductopenia and perivenous fibrosis as well [113]. Several studies have shown a connection between the length of PN and the severity of liver damage [114,115,116].

## 6. Management

### 6.1. Optimizing Nutrition

#### 6.1.1. Duration of Nutrition

A retrospective study on 107 patients with gastroschisis showed that prophylactic cyclic PN is associated with a lower incidence and later onset of hyperbilirubinemia. This means that patients with IF, especially those who require long-term PN, should take into account cyclic PN infusion (<24 h, often 8–12 h) [117]. The advantages of initiating prophylactic cyclic PN in surgical neonates include decreased risk of hyperbilirubinemia, lower levels of conjugated bilirubin, less frequent use of bilirubin-lowering drugs, and shorter length of stay in the hospital. Early initiation of prophylactic cyclic PN does not enhance the likelihood of adverse outcomes [118]. Cyclic PN infusion may stabilize liver function tests in certain patients receiving continuous PN infusion and mild hyperbilirubinemia (< or =20 ng/mL). Abrupt infusion onset may cause hyperglycemia, while abrupt discontinuation may cause hypoglycemia, most commonly in children under the age of 2–3 years [119].

#### 6.1.2. Composition of Nutrition

A prospective randomized control trial showed that infants at risk of IFALD taking low-dose SOLE (1 g/kg/day) had a slower rate of increase of cholestasis markers compared to those taking the standard dose (3 g/kg/day) [120]. In contrast, a multicenter randomized controlled trial showed that low-dose SOLE (1 g/kg/day) was not associated with a reduction in cholestasis or growth [121]. These different conclusions might be due to differences in the population sample, such as the gestational age of the infants, the inclusion of infants with intestinal disease and surgical procedures, and the age when beginning PN.

FOLE monotherapy has been shown to have a better rate of cholestasis resolution when compared with SOLE [42,122]. A multicenter retrospective study demonstrated that FOLE recipients had higher prealbumin, lower triglyceride, and more normal glucose concentrations compared with SOLE recipients. There were no growth differences between the groups [41]. In patients who received SOLE, FOLE helped in decreasing conjugated bilirubin, leading to cholestasis resolution. However, switching back to SOLE led to rebound hyperbilirubinemia, which is challenging to treat [123]. A double-blind randomized controlled trial showed that the progression of IFALD can be stopped by replacing SOLE with FOLE and reversed by increasing the EN in infants on FOLE [124]. FOLE as monotherapy with a daily dose of 1 g/kg has been shown to prevent EFAD in PN-dependent patients [125]. Studies have shown that FOLE is not associated with EFAD, bleeding, or other complications [126,127].

Olive oil-based lipid emulsion (OOLE) was shown to be well tolerated in critically ill neonates in a randomized double-blind study. Plasma phospholipid oleic acid were increased and linoleic acid levels were decreased in patients on OOLE versus patients on SOLE [128]. FOLE combined with OOLE delayed the onset of IFALD in premature neonates with SBS [129].

Another study showed that MOLE is more hepatoprotective than SOLE, demonstrating that long term exposure (>4 weeks) to MOLE lead to lower conjugated bilirubin than SOLE [130]. In a retrospective cohort study in 107 patients, IFALD developed in 44.8% of patients receiving SOLE compared with 30% of patients receiving MOLE; in the multivariable analysis, it was found that the type of ILE was not a risk factor, while the duration of PN and lipids were [131].

#### 6.1.3. Advancing Enteral Feedings

The early introduction of enteral feeding and subsequent progression to full feeding improves outcomes in patients with IF and reduces the risk of cholestasis. In a cohort study, administering greater initial EN volumes (20 mL/kg/d) and daily feeding advancement resulted in a 60% reduction in the likelihood of developing IFALD of any severity and a 72% reduction in the likelihood of developing moderate-to-severe IFALD [132]. Enteral feeding decreases the risk of cholestasis through various mechanisms, including stimulation of bile flow and gallbladder contraction, increasing intestinal motility, induction of gut hormone secretion, and promotion of mucosal hyperplasia/hypertrophy. These factors collectively contribute to intestinal adaptation [133,134].

### 6.2. Pharmacologic Therapies

#### 6.2.1. Ursodeoxycholic Acid

Ursodeoxycholic acid (UDCA) is a multifunctional therapeutic agent used to treat cholestatic hepatopathies. UDCA prevents cytolysis and apoptosis, changes the expression of enzymes and transporters that reduce bile acid cytotoxicity, modulates ductular bile flow, prevents endocytic internalization of canalicular transporters, and has immunomodulatory properties [135]. UDCA has been shown to improve the biochemical and clinical signs and symptoms of IFALD. Studies on adults and infants with SBS and IFALD have shown association between improved hyperbilirubinemia and UDCA administration. UDCA treatment has been found to reduce the levels of GGT, ALT, ALP, and direct bilirubin, leading to resolution of cholestasis within two months [136,137,138,139]. In a prospective double-blind placebo-controlled trial, infants who received UDCA had a steady reduction in GGT and a significant reduction in AST and ALT. However, their serum bilirubin levels remained similar compared to the control group [140]. Another prospective double-blind randomized controlled trial in very low birth weight infants showed that UDCA was most successful at preventing cholestasis while oral erythromycin was most effective at facilitating enteral feeding [141].

#### 6.2.2. Cholecystokinin-Octapeptide (CCK-OP)

A multicenter double-blind randomized controlled trial on 243 neonates showed that the use of CCK-OP did not significantly affect conjugated bilirubin levels or the incidence of IFALD. This provides evidence against the administration of CCK-OP in patients with IFALD [142].

#### 6.2.3. Intestinal Growth Factors

In a 24 week prospective study, teduglutide (a glucagon-like peptide 2 analogue) was shown to reduce the number of days and volume of parenteral support for patients with SBS with IF [143]. This is supported by the results other cohort studies [144,145,146,147,148]. In addition to the reduction in PN, there improved stool frequency and consistency has been found [149,150]. Significant reductions in parenteral support were seen in a pediatric population with SBS with IF over 24 weeks in a phase II study of teduglutide [151]. The intestinal anatomy has been found to strongly influence outcomes, leading to the hypothesis that teduglutide-induced changes might persist in the pediatric population even after stopping treatment because of the continuous growth of the intestine [152]. Even though teduglutide shows great potential for reducing the occurrence of IFALD, as it reduces the need for PN and promotes enteral autonomy, further research is necessary in order to assess its long-term efficacy, effects after discontinuation, and potential complications. Glepaglutide was shown to improve hepatic excretory function in a phase 2 clinical trial, although it also activated liver macrophages and increased liver stiffness [153]. Another GLP-2 analogue, apraglutide, has been shown to improve intestinal adaptation in SBS neonatal piglets [154]. GLP-1 analogues such as liraglutide have been researched in patients with SBS-IF and jejunostomies, with results showing that it can reduce ostomy wet weight output and increase intestinal wet weight and energy absorption [155]. GLP-1/GLP-2 co-agonists are currently being researched on animal models, showing effects on gut morphometry, increased intestinal volume, and increased mucosal surface area [156].

#### 6.2.4. Phenobarbital

A case report on a premature infant on PN showed that phenobarbital reduced bilirubin and hepatic enzyme levels [157]. However, in a retrospective study on neonates receiving PN phenobarbital was not effective in preventing IFALD [158]. In animal models phenobarbital showed neurotoxic effects such as apoptotic neurodegeneration in white matter and impaired associative memory or recall [159,160]. This provides evidence against the administration of phenobarbital in patients with IFALD.

#### 6.2.5. Metronidazol

Based on the involvement of anaerobic intestinal bacteria in the pathogenesis of IFALD, a retrospective study investigated the effect of metronidazole on IFALD and showed prevention of the expected increase in ALP, GGT, and AST [161]. In patients with Crohn’s disease, the administration of metronidazole helped to reduce or maintain normal levels of live enzymes after 30 days of total PN [162]. Despite the potential of bacterial translocation to contribute to hepatocyte injury, the Pediatric Surgical Association Outcomes and Clinical Trials Committee has declared that there are insufficient data for the routine use of antibiotics such as metronidazole in the prevention of IFALD [163].

#### 6.2.6. Erythromycin

Erythromycin has been researched as a prokinetic agent in promoting enteral feeding in preterm infants with feeding intolerance [164]. High-dose oral erythromycin (12.5 mg/kg/dose every 6 h for 14 days) in preterm infants on PN has been shown to reduce the duration of PN and incidence of IFALD, and to lead to full EN significantly earlier [165]. Intermediate-dose oral erythromycin (5 mg/kg oral erythromycin every 6 h for 14 days) helped in reducing the incidence of IFALD and necrotizing enterocolitis in infants on PN by reducing the duration of PN and the days required to achieve full enteral feeding [166]. Erythromycin has been shown to be significantly more effective in facilitating full enteral feeding compared to UDCA [141].

### 6.3. Microbiome Therapies

Antibiotic treatment has been used therapeutically in combination with probiotics. In a 22-year-old patient with SBS, D-lactate producing Lactobacili were eliminated using kanamycin 400 mg/d, then the bowel was recolonized with nonpathogenic flora with the help of probiotics containing *Bifidobacterium breve* and *Lactobacillus casei* [167]. In a patient with SBS and neurologic dysfunction due to D-lactic acidosis, antibiotic treatment was ineffective, while a synbiotic treatment with *Bifidobacterium breve Yakult* and *Lactobacillus casei Shirota* as probiotics and galacto-oligosaccharide as a prebiotic led to remission of D-lactate acidosis [168]. In a case report involving a 7-year-old child, fecal microbiota transplantation was demonstrated to be effective in the treatment of D-lactic acidosis [87].

### 6.4. Prevention of CLABSI/Sepsis

Proper prevention includes antisepsis with chlorhexidine on the insertion place, utilization of antimicrobial catheter locks and coatings, adherence to maximum sterile barrier precautions, and regular disinfection of hubs and connectors [169]. Ethanol locks were shown to reduce the CLABSI rate by 81% in pediatric IF patients, while the use of taurolidine-citrate locks reduced it by 92% compared to saline locks [170,171]. In cases where CLABSI is suspected, it is essential to obtain a catheter culture and promptly initiate empirical antibiotic therapy accordingly [172].

### 6.5. Surgical Lengthening Procedures

Small bowel length, an intact ileocecal valve, intestinal continuity, and preservation of the colon are crucial factors for survival and adaptation in SBS patients on PN [173]. Surgical lengthening procedures are important in patients with SBS and IF for optimizing the absorptive surface of the gut and reducing the need for PN. In a retrospective study, longitudinal intestinal lengthening and tailoring (LILT) and serial transverse enteroplasty (STEP) were shown to be feasible for reaching enteral autonomy and weaning of PN in 59% of adult patients [174]. LILT increases peristalsis, reduces bacterial overgrowth, and improves nutrient absorption by lengthening the mucosal contact time. In a cohort study, all patients with liver fibrosis who underwent LILT showed normalization of AST, ALT, and bilirubin, and were successfully weaned from PN [175]. A case report on a 2-year-old showed that STEP increased intestinal length and can improve intestinal absorptive capacity [176].

### 6.6. Transplantation

Transplantation is indicated in children with life -threatening complications of IF and PN, such as severe IFALD. In cases where patients exhibit no or minimal signs of IFALD, isolated small bowel transplantation is a potential consideration; however, for severe IFALD cases characterized by fibrosis or cirrhosis the most commonly performed procedure is a combined liver and intestine transplant [177]. Transplantation has been shown to be better for re-establishing nutritional autonomy and to be cost-effective [178]. Survival rates for patients differed based on age and the type of transplant received, with pediatric intestine recipients having the highest survival rates (1-year survival of 86.2% and 5-year survival of 75.4%) and adult intestine–liver recipients having the lowest (1-year survival of 66.7% and 5-year survival of 42.6%) [179]. Intestinal transplants are significantly immunogenic due to the quantity of lymphoid tissue, high cellular turnover, and numerous intraluminal bacteria. This leads to a higher risk of developing infection, acute allograft rejection, kidney disease, lymphoproliferative disorders, and GVHD [180]. In addition to the standard desensitization protocol, different strategies for counteracting donor-specific antibodies have been researched, such as the incorporation of rituximab and bortezomib, belatacept and bortezomib, or eculizumab and a C1 esterase inhibitor. With further advancements in surgical techniques and post-operative care, including immunosuppression, these results should continue to improve in the future [177,180].

## 7. Conclusions

The full spectrum of IFALD continues to be a serious complication in pediatric and adult patients on long-term PN. Its etiology is multifactorial, consisting of different variables that can be either PN-related (nutrient toxicity and nutrient deficiency) or patient-related (related to IF and systemic factors). The perfect non-invasive diagnostic tool for IFALD has not yet been established, and liver biopsy continues to be the most effective method for diagnosing and tracking its development. The management of IFALD is multidisciplinary and should be individualized for each patient. This may consist of optimizing nutritional strategy, using pharmacologic and microbiome therapies, preventing CLABSI and sepsis, and providing surgical care such as surgical lengthening procedures and transplantation. Additional research is needed in order to develop more targeted metabolomics approach and possible treatment drugs based on the pathogenesis of IFALD, as well as further research on improving the formulas used in PN.

## Figures and Tables

**Table 1:** Eligibility criteria for study selection.

Inclusion Criteria	Exclusion Criteria
Adult and pediatric patients Patients on PN Patients with IFALD English language	Reviews Letters to the editor Opinion pieces

**Table 2:** Factors involved in the etiology of IFALD in adult and pediatric populations.

Risk Factor Category	Adult Population	Pediatric Population
PN related factors	Nutrient toxicity	
	- Excessive energy intake - Glucose overload (>7 mg/kg/min) - Lipid emulsion (LCT) overload - Soybean LE (>1 g/kg/d) - Continuous infusion (24/7 h) - Phytosterols - Trace elements overload	- Excessive energy intake - Soybean LE (>1 g/kg) - Glucose overload (>7 mg/kg/min) - Phytosterols - Trace elements overload
	Nutrient deficiency	
	- Antioxidant deficiency - Deficiency of choline, carnitine, methionine, taurine, essential fatty acid deficiency, vitamin C, vitamin E	- Deficiency of choline - Essential amino-acid deficiency - Prolonged period without EN and prolonged PN use
Patient related factors	IF-related	
	- Lack of oral feeding - Presence of SBS - Small bowel bacterial overgrowth - Microbiome - Suppression of Paneth cell bactericidal response - Decreased IgA secretion - Increased translocation of endotoxins and bacteria - Alteration of bile acid metabolism - Loss of gut hormone stimulation - Decrease of FGF19 levels	- Long duration of PN that leads to GI imbalances/dysfunction - Fasting or low enteral intake - Presence of SBS with stoma - Presence of gastroschisis - Presence of intestinal atresia - Intestinal and biliary stasis - Small bowel bacterial overgrowth and intestinal dysbiosis
	Systemic-related	
	- Central line-associated blood stream infections - Infection, sepsis - Influence of hepatotoxic agents such as alcohol consumption, drugs, viral hepatitis, and autoimmune liver disease	- Prematurity - Low birth body weight - Initiation of PN at a young age - Infection/Frequent sepsis - Medications - Genetic predisposition - Central line-associated blood stream infections

Table 2 was created by including relevant articles and adapted from the following references within the field: [6,22,23,24,25,26,27,28,29,30,31,32,33,34,35,36].

## Data Availability

Data sharing not applicable.

## Abbreviations

ALA	α-linoleic acid
CCK-OP	cholecystokinin-octapeptide
CLABSI	central line-associated blood stream infections
EFAD	essential fatty acid deficiency
EN	enteral nutrition
FOLE	fish oil-based intravenous lipid emulsion
IF	intestinal failure
IFALD	intestinal failure-associated liver disease
ILE	intravenous lipid emulsion
LA	linoleic acid
LCT	long chain triglycerides
LILT	longitudinal intestinal lengthening and tailoring
LPS	lipopolysaccharides
MCT	medium chain triglycerides
MOLE	mixed-oil or composite intravenous lipid emulsion
MRS	magnetic resonance spectroscopy
MUFA	monounsaturated fatty acids
NAFLD	non-alcoholic liver disease
OOLE	olive oil-based intravenous lipid emulsion
PN	parenteral nutrition
PNAC	parenteral nutrition-associated cholestasis
PNALD	parenteral nutrition-associated liver disease
PUFA	polyunsaturated fatty acids
SBS	short bowel syndrome
SIBO	small intestine bacterial overgrowth
SOLE	soybean oil-based intravenous lipid emulsion
STEP	serial transverse enteroplasty
TE	transient elastography
UDCA	ursodeoxycholic acid
